# Involvement of Lipid Rafts in the Invasion of Opportunistic Bacteria *Serratia* into Eukaryotic Cells

**DOI:** 10.3390/ijms24109029

**Published:** 2023-05-20

**Authors:** Yuliya Berson, Sofia Khaitlina, Olga Tsaplina

**Affiliations:** Institute of Cytology, Russian Academy of Sciences, Tikhoretsky av. 4, 194064 St. Petersburg, Russia; juletschka.ber@gmail.com (Y.B.); skhspb@gmail.com (S.K.)

**Keywords:** lipid rafts, bacterial invasion, EGFR, *Serratia*

## Abstract

Cell membrane rafts form signaling platforms on the cell surface, controlling numerous protein–protein and lipid–protein interactions. Bacteria invading eukaryotic cells trigger cell signaling to induce their own uptake by non-phagocytic cells. The aim of this work was to reveal the involvement of membrane rafts in the penetration of the bacteria *Serratia grimesii* and *Serratia proteamaculans* into eukaryotic cells. Our results show that the disruption of membrane rafts by MβCD in the three cell lines tested, M-HeLa, MCF-7 and Caco-2, resulted in a time-dependent decrease in the intensity of *Serratia* invasion. MβCD treatment produced a more rapid effect on the bacterial susceptibility of M-HeLa cells compared to other cell lines. This effect correlated with a faster assembly of the actin cytoskeleton upon treatment with MβCD in M-HeLa cells in contrast to that in Caco-2 cells. Moreover, the 30 min treatment of Caco-2 cells with MβCD produced an increase in the intensity of *S. proteamaculans* invasion. This effect correlated with an increase in EGFR expression. Together with the evidence that EGFR is involved in *S. proteamaculans* invasion but not in *S. grimesii* invasion, these results led to the conclusion that an increase in EGFR amount on the plasma membrane with the undisassembled rafts of Caco-2 cells after 30 min of treatment with MβCD may increase the intensity of *S. proteamaculans* but not of *S. grimesii* invasion. Thus, the MβCD-dependent degradation of lipid rafts, which enhances actin polymerization and disrupts signaling pathways from receptors on the host cell’s surface, reduces *Serratia* invasion.

## 1. Introduction

The cells of all living organisms are bounded by membranes that support the shape of the cell and define the boundary between the inside of the cell and the outside environment. The membranes are formed by a lipid bilayer in which the hydrophobic properties of lipids contribute to their association with each other, and the hydrophilic properties of proteins located between the lipid layers determine their interaction with aqueous media and with each other [1]. At the same time, membranes are capable of lateral segregation of lipid and protein components, which makes it possible to coordinate their numerous functions. This ability, based on the fact that lipid and protein components do not mix, underlies the concept of lipid rafts, which provide subcompartmentalization of the cell membrane [2,3,4,5]. Rafts are heterogeneous, dynamic, cholesterol- and sphingolipid-enriched membrane nanodomains 10–200 nm in size that can form larger domains (>300 nm) upon protein-induced clustering. Rafts interact with the underlying cytoskeleton and are a platform for such processes as the transport, sorting, signaling and penetration of pathogenic and opportunistic bacteria [5,6,7,8,9,10]. 

The penetration of both pathogenic and opportunistic bacteria into eukaryotic cells (invasion) is a process initiated by the contact of bacteria with an eukaryotic cell and occurs according to one of the two currently known mechanisms: trigger or zipper [11]. Bacteria that penetrate eukaryotic cells using a trigger mechanism inject virulence factors directly into the cytoplasm of host cells through a special secretion apparatus, thereby triggering rearrangements of the cytoskeleton and the formation of protrusions, leading to the internalization of bacteria [11,12,13]. Bacteria that enter eukaryotic cells using the zipper mechanism promote the interaction of their specific surface proteins with host cell receptors. This activates the host cell’s signaling systems and induces cytoskeleton rearrangements whereby the membrane bends around the bacteria and closes like a zipper which leads to the absorption of bacteria [14]. Thus, bacterial invasion into eukaryotic cells is strongly associated with the contact of bacteria with the host cell’s surface proteins including those in membrane lipid rafts containing cell receptors, proteins of the signal transduction system, kinases and other proteins necessary for the normal activity of eukaryotic cells [15].

The capability of opportunistic bacteria to penetrate eukaryotic cells raises the question of the role of membrane rafts in the invasive activity of these bacteria. The opportunistic bacteria *Serratia grimesii* and *Serratia proteamaculans* penetrate into eukaryotic cells and are found in vacuoles and, after leaving the vacuole, in the cytoplasm [16,17,18,19,20,21]. Our electron microscopy data suggested that the first stage of *S. grimesii* invasion is the interaction of the bacterial invasin with the surface receptors of the host cell [20]. One of these receptors seems to be E-cadherin [22,23,24]. However, the mechanisms of the further penetration of these bacteria into eukaryotic cells remain unclear. Therefore, the aim of this work was to reveal the involvement of membrane rafts in the penetration of the bacteria *S. grimesii* and *S. proteamaculans* into eukaryotic cells. To this end, we compared the invasion of *S. grimesii* and *S. proteamaculans* into cells whose membrane rafts were destroyed through the pretreatment of these cells with methyl-β-cyclodextrin (MβCD) [25].

## 2. Results

**Figure 1:**
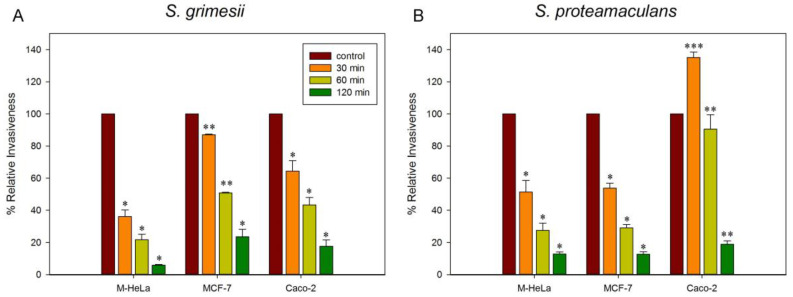
Effect of methyl-β-cyclodextrin (MβCD) on *S. grimesii* (**A**) and *S. proteamaculans* (**B**) invasion into M-HeLa/MCF-7/Caco-2 cells. Eukaryotic cells were treated with 5 mM MβCD for the indicated time before incubation with bacteria. Control: untreated cells. The number of intracellular bacteria was estimated as a percentage, taking the number of intracellular bacteria in control samples as 100%. Values are expressed as mean S.D. (error bars). A difference to the control was considered significant at the * *p* < 0.05, ** *p* < 0.001 and *** *p* < 0.0001 levels.

After the treatment of cultured eukaryotic cells with MβCD, we quantified their sensitivity to *S. grimesii* and *S. proteamaculans* invasion. We have previously shown that the treatment of eukaryotic cells with the antioxidants N-acetylcysteine and dihydrolipoic acid, as well as with a selective inhibitor of Rho-associated protein kinase (ROCK) which increases the amount of E-cadherin, also increases cell sensitivity to *S. grimesii* [22,23,24]. Therefore, in this work, we quantified the effect of MβCD on the sensitivity of cervical carcinoma M-HeLa cells, which normally do not synthesize E-cadherin [26], colorectal adenocarcinoma epithelial Caco-2 cells, and breast cancer MCF-7 cells, which should synthesize E-cadherin characteristic of epithelial cells [27], to opportunistic bacteria *S.grimesii* and *S.proteamaculans*. At 100 bacteria per cell, the intensity of *S. grimesii* invasion is 2 times higher than that of *S. proteamaculans*. The sensitivity of M-HeLa and MCF-7 cells to these bacteria is the same, and 4-5 times more bacteria enter Caco-2 cells during incubation. To determine the effect of MβCD on the sensitivity of cells to bacteria, we normalized the intensity of invasion after the incubation of cells with MβCD to the intensity of bacterial invasion into untreated cells. The decrease in the intensity of invasion depends on the time of treatment with MβCD. Apparently, the extraction of cholesterol continued for 2 h of incubation with MβCD. The dynamics of the decrease in the invasion of *S. grimesii* and *S. proteamaculans* into the cells pretreated with MβCD was different both in cells of a different origin and in different bacteria (Figure 1). Cyclodextrin-dependent cholesterol removal reduced the *S. grimesii* invasion of M-HeLa cells more than that of MCF-7 and Caco-2 cells (Figure 1A). The dynamics of *S. proteamaculans* invasion into the cells pretreated with MβCD decreased similarly for M-HeLa and MCF-7 cells. However, unexpectedly, a 30 min incubation with MβCD sufficiently increased the sensitivity of Caco-2 cells to bacteria by 35% (Figure 1B). We used Western blot analysis and assessed the distribution of EGFR on the cell surface using confocal microscopy. However, the sensitivity of these methods did not allow one to obtain statistically significant changes in the amount of EGFR in Caco-2 cells after 30 min of MβCD treatment.

**Figure 2:**
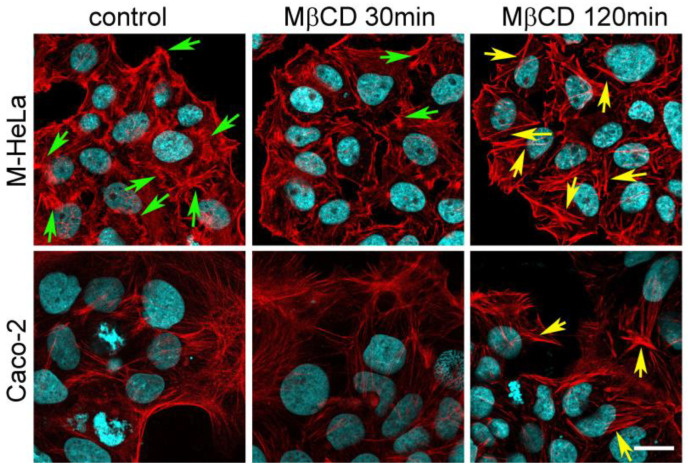
Cytoskeleton of eukaryotic cells treated with methyl-β-cyclodextrin. After 30 min and 120 min incubation of M-HeLa and Caco-2 cells with MβCD, actin was stained with rhodaminephalloidin, and the nuclei were stained with DAPI. Protrusions and stress fibrils are indicated with green and yellow arrows, respectively. Control: untreated cells. Scale: 20 µm.

Rearrangements of the actin cytoskeleton are involved in both the trigger and the zipper mechanism of bacterial invasion [11]. We have previously shown that the treatment of eukaryotic cells with MβCD could lead either to the assembly or disassembly of the actin cytoskeleton, depending on its initial state in the cells [28]. Therefore, we compared whether MβCD induces rearrangements of the actin cytoskeleton in the cells in which MβCD treatment causes a decrease (M-HeLa) or an increase (Caco-2) in sensitivity to *S. proteamaculans*. Using confocal microscopy, we showed that after 30 min of incubation, MβCD promotes the disappearance of protrusions (indicated by green arrows) along the cell perimeter and changes the shape of M-HeLa cells (Figure 2, top row). After 2 h of incubation, M-HeLa and Caco-2 cells revealed a significant amount of stress fibers (Figure 2). This explains the faster effect of MβCD on bacterial entry into M-HeLa cells than into Caco-2 cells. However, different effects of MβCD on the sensitivity of M-Hela/MCF-7 and Caco-2 cells to *S. proteamaculans* cannot be caused by cytoskeletal rearrangements.

**Figure 3:**
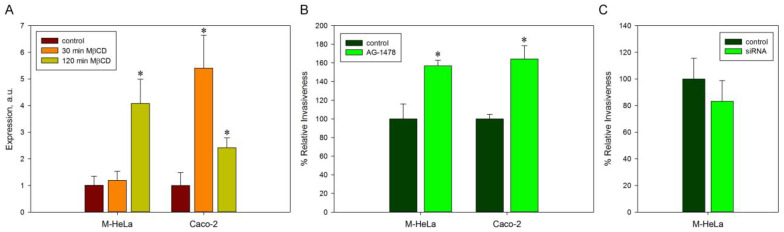
EGFR in host cells. (**A**) EGFR expression in M-HeLa and Caco-2 cells after 30 min and 120 min incubation with MβCD was assessed using real-time RT-PCR. Control: untreated cells. (**B**) Effect of an EGFR inhibitor (tyrphostin AG-1478) on the sensitivity of M-HeLa and Caco-2 cells to *S. grimesii* invasion. Control cells were incubated in DMEM with the addition of 0.1% ethanol. The number of intracellular bacteria was estimated as a percentage, taking the number of intracellular bacteria in control samples as 100%. Values are expressed as mean S.D. (error bars). A difference to the control was considered significant at the * *p* < 0.05 level. (**C**) Effect of treating M-HeLa cells with siRNA targeting EGFR on cell sensitivity to *S. grimesii* invasion. Control: M-HeLa cells transfected with siRNA containing scrambled nucleotide sequence. Cell pretreatment with small interfering RNA reduced gene expression and amount of EGFR (Figure S1).

Previously, we showed that the epidermal growth factor receptor (EGFR), which is located in the lipid rafts of the host cell, is involved in the invasion of *S. proteamaculans* [29]. Therefore, using real-time RT-PCR, we tested whether MβCD treatment had an effect on the expression of the EGFR gene. The treatment with MβCD increased the expression of the EGFR in M-HeLa cells only after 2 h of incubation and already after 30 min of incubation in Caco-2 cells (Figure 3A). Thus, the increase in the EGFR expression as a result of the 30 min incubation of host cells with MβCD may contribute to an increase in *S. proteamaculans* invasion. 

To determine whether EGFR is involved in *S. grimesii* invasion, we used the selective EGFR inhibitor tyrphostin AG-1478. Previously, we have shown that both tyrphostin AG-1478 and siRNA targeting EGFR reduce the intensity of *S. proteamaculans* invasion by 30% [29]. Treatment of M-HeLa and Caco-2 cells with an EGFR inhibitor increased the invasion of *S. grimesii* into these cells by 1.5 times (Figure 3B). In order to rule out the nonspecific effect of tyrphostin AG-1478, we evaluated the effect of cell treatment with siRNA targeting EGFR under previously tested conditions [29] on *S. grimesii* invasion into M-HeLa cells. Pretreatment of M-HeLa cells with siRNA targeting EGFR reduces the sensitivity of cells to *S. grimesii* by only 15% (Figure 3C). Thus, the EGF receptor is not a major player in *S. grimesii* invasion, and the increase in EGFR expression resulting from MβCD treatment may not promote *S. grimesii* invasion.

**Figure 4:**
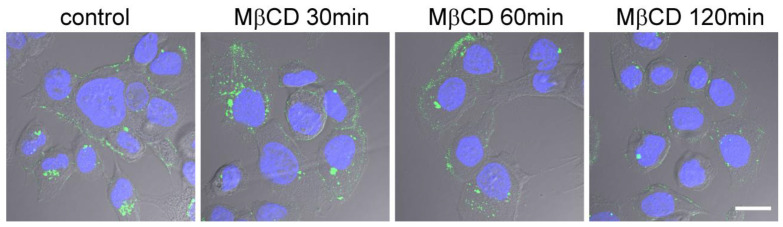
Redistribution of rafts upon treatment with methyl-β-cyclodextrin. After 30, 60 and 120 min of Caco-2 cells’ incubation with MβCD, cholesterol was stained with the B subunit of cholera toxin (CTxB-FITC), and nuclei were stained with DAPI. Cell shape was assessed using phase contrast (grayscale). Control: untreated cells. Scale: 20 µm.

EGFR is located in rafts, and the destruction of these rafts would impair EGFR functions [30,31]. Therefore, an increase in EGFR expression can affect the sensitivity of treated cells to bacteria only if the rafts remain on the surface of the host cell. We evaluated the effect of MβCD on cholesterol distribution in Caco-2 cells. For this, cholesterol was labeled with the cholera toxin subunit B (CTxB) conjugated with FITC (Figure 4). After 30 min of incubation with MβCD, there is no decrease in cholesterol along the perimeter of Caco-2 cells, which is observed after 2 h of incubation. Thus, an increase in EGFR expression can lead to an increase in the intensity of *S. proteamaculans* invasion after 30 min of a host cell’s incubation with MβCD, whereas, after 2 h of incubation, the destruction of rafts already plays a key role in determining the intensity of invasion.

## 3. Discussion

Lipid rafts are heterogeneous cell membrane nanodomains enriched in cholesterol and sphingolipids which are capable of clustering due to protein–protein and protein–lipid interactions, thus forming functional platforms for the regulation of cellular processes [3,5,32,33]. The stability of rafts is determined by the presence of cholesterol in their composition [34]. Therefore, a common way to reveal the role of lipid rafts in cell life is the destruction of the rafts by removing cholesterol [25,35,36]. Most often, this result is achieved by incubating cells with MβCD or other cyclodextrins [25]. The cholesterol binding agents (digitonin and saponin) are not compatible with the live cells because they are detergents. The filipin is also not compatible with the live cells because it forms crystals with immobilized cholesterol resulting in leaky membranes [36]. MβCD has a central cavity able to form a 2:1 complex with cholesterol [37]. This complex does not produce degradation products and is compatible with live cell studies [36]. Moreover, MβCD has the advantage of acting strictly at the membrane surface [37]. The cholesterol concentration in the plasma membrane of mammalian cells is generally around 35–45 mol% of total lipids. In normal cholesterol depletion experiments, the cholesterol concentration is typically decreased by only 40% to 20–27 mol% [38]. MβCD-dependent cholesterol extraction may regulate endocytosis through effects on cell membrane fluidity and lipid phase order [39]. However, model experiments on lipid membranes indicate that in receptor-independent endocytosis, treatment of MβCD cells should increase bacterial uptake rather than inhibit it, as in our experiments [39]. Therefore, it is of interest that despite the presence of high concentrations of cholesterol remaining in the “cholesterol-depleted” plasma membrane, many signaling reactions are shut down [38]. MβCD treatment enhances EGFR autophosphorylation 2–5-fold in the first 5 min of incubation and also inhibits the down-regulation of EGFR. This likely contributes to the enhanced ability of EGFR to stimulate downstream signaling pathways [40].

Lipid rafts have been reported to be involved in the entry of various bacterial pathogens and opportunistic pathogens into non-phagocytic cells [8,9,31,41,42,43]. In our work, we compared the effects of raft disruption on the sensitivity of eukaryotic cells of various origins to the invasion of opportunistic bacteria *S. grimesii* and *S. proteamaculans*. We have previously shown that *S. proteamaculans* synthesize several virulence factors [44]. The intensity of their invasion can be determined by the actin-specific protease protealysin, hemolysin ShlA, and the extracellular protease serralysin [44]. At the same time, in *S. grimesii*, only the actin-specific protease grimelysin, which can provide the rearrangements of the cytoskeleton necessary for penetration into the host cell, was found [44]. Invasion-promoting toxins have been found to bind to lipid rafts that promote subsequent oligomerization at the host cell’s surface [41,45]. For efficient channel formation, these toxins require molecules with a high affinity for rafts [41,46]. However, the hemolysin ShlA of *Serratia* does not contain a cholesterol-binding domain [47]. The membrane binding and insertion of ShlA are highly dependent on phosphatidylserine, which targets the toxic activity to eukaryotic cell membranes without any need of a proteinaceous receptor [48]. Thus, the different effects of treatment with MβCD on the sensitivity of cells to invasion by *S. grimesii* and *S. proteamaculans* cannot be due to the fact that only *S. proteamaculans* synthesizes hemolysin ShlA.

Rafts control numerous protein–protein and lipid–protein interactions at the cell surface. This function is possible due to two important properties of the rafts: their ability to selectively incorporate or exclude proteins and their ability to combine into larger domains. Upon ligand binding, lipid rafts in the “off state” become activated and form clusters to generate a platform of rafts in the “on state” in which all the components required for signaling are in close proximity. The raft binding recruits proteins to a new micro-environment where the phosphorylation state can be modified by local kinases and phosphatases, resulting in downstream signaling [49]. On the other hand, it is well established that pathogens trigger signaling particularly when they induce their own uptake by non-phagocytic cells. It has therefore been speculated that one of the attractive aspects of lipid rafts for bacteria is this ability to efficiently signal because of the capacity to bring molecules involved in signaling together [43]. First, lipid rafts were shown to be important for the initial clustering of E-cadherin molecules during multivalent binding to internalin A present on bacteria [42]. In contrast, cholesterol depletion did not affect internalin B binding to HGF-R or HGF-R recruitment at the plasma membrane. However, downstream signaling events required membrane cholesterol because internalin B-induced membrane ruffles and actin-rich phagocytic cups were not observed after cholesterol depletion [42]. In addition, lipid rafts have been shown to be involved in a variety of signaling mechanisms including B-cell receptor, T-cell receptor, EGF, and integrin-mediated signaling [49]. The pretreatment of HeLa cells with MβCD disrupts *Campylobacter jejuni*-dependent EGFR phosphorylation [30]. The binding of EGF by EGFR in lipid rafts triggers receptor dimerization and autophosphorylation, which leads to the activation of multiple signaling proteins [31]. MβCD disrupts the interaction between EGFR and the β1 integrin, which explains the mechanistic basis for the inhibition of *C. jejuni* internalization [30]. Furthermore, the α5β1 integrin, independent of lipid rafts, promotes *Porphyromonas gingivalis* adhesion to epithelial cells, while the subsequent uptake process requires lipid raft components for actin organization with Rho GTPase Rac1 [50]. Thus, the different effect of raft disassembly on the cells of different cell lines can be due to the number of receptors required for invasion on the host cell’s surface before the MβCD treatment.

The disassembly of rafts has a different effect on the dynamics of increasing sensitivity to *S. grimesii* and *S. proteamaculans* invasion. In this work, we found that EGFR, which plays a significant role in the invasion of *S. proteamaculans* [29], is not involved in the invasion of *S. grimesii*. Thus, the difference in the amount of EGFR on the surface of the cells of different cell lines used may explain the different effect of MβCD treatment on cell sensitivity to *S. grimesii* and *S. proteamaculans*. Treatment with MβCD can affect gene expression in eukaryotic cells. Genes coding for proteins related to adherens junctions, focal adhesion, endocytosis, the regulation of autophagy and the regulation of actin cytoskeleton were up-regulated after MβCD treatment [51]. In this work, we showed that treatment with MβCD increased the expression of EGFR in M-HeLa cells only after 2 h of incubation, while, in Caco-2 cells, EGFR expression already increased after 30 min of incubation. Thus, the increase in the EGFR expression as a result of the 30 min incubation of Caco-2 cells with MβCD may lead to an increase in the amount of EGFR on the host cell’s surface and enhance *S. proteamaculans* invasion. Therefore, the treatment of Caco-2 cells with MβCD resulting in increased EGFR expression does not increase EGFR-independent *S. grimesii* invasion.

Membrane cholesterol and lipid rafts are implicated in various signaling processes involving actin rearrangement in living cells. Actin is the most important protein that plays a key role in the entry of bacteria into eukaryotic cells [11]. Previously, we have shown that the effects of cholesterol depletion and lipid raft disruption on the microfilament system is critically determined by the initial state of the cytoskeleton; specifically, it is determined by the balance of polymerized and monomeric actin in the cell [28]. In this work, we showed that MβCD promotes an increase in the number of stress fibers in M-HeLa cells as early as 30 min after incubation and only after 2 h of incubation in Caco-2 cells. Bacterial invasion of non-phagocytic cells requires remodeling of the actin cytoskeleton to form actin-rich cell surface projections designed to engulf the bacteria [11]. Apparently, an increase in the number of actin fibers leads to a decrease in the sensitivity of eukaryotic cells to bacteria because fibrillar actin must first depolymerize in order to polymerize later into the structures needed for bacterial uptake. Thus, the different incubation time with MβCD required for the formation of stress fibers may explain the faster effect on the susceptibility of M-HeLa cells to bacteria compared to that of Caco-2 cells. Thus, the MβCD-dependent degradation of lipid rafts, which promotes actin polymerization and disrupts signaling pathways from receptors on the host cell’s surface, reduces *Serratia* invasion.

## 4. Materials and Methods

### 4.1. Cell Cultures, Bacterial Strains and Growth Conditions

The cervical carcinoma M-HeLa, breast adenocarcinoma MCF-7 and colorectal adenocarcinoma Caco-2 cell lines were obtained from the “Vertebrate cell culture collection” (Institute of Cytology, St. Petersburg, Russia) supported by the Ministry of Science and Higher Education of the Russian Federation (Agreement 075-15-2021-683). Cells were grown in DMEM (Sigma-Aldrich, Munich, Germany) and 10% fetal bovine serum (Sigma-Aldrich, Munich, Germany). 

*Serratia proteamaculans* strain 94 was isolated as described earlier [52]. *Serratia grimesii* strain 30063 was obtained from the German Collection of Microorganisms and Cell Cultures (DSMZ). *S. proteamaculans* and *S. grimesii* were grown in LB medium (Sigma-Aldrich, Munich, Germany) at 30 and 37 °C, respectively, with aeration. Previously, we have shown that the invasion of *S. grimesii* and *S. proteamaculans* correlates with the synthesis of actin-specific proteases grimelysin and protealysin, respectively, which are activated at the stationary stage of bacterial growth [17,18,19]. In preliminary experiments, we evaluated the sensitivity of M-HeLa cells to *Serratia* after 24 and 48 h of growth. We have shown that the sensitivity of M-HeLa cells to *S. proteamaculans* invasion after 24 and 48 h of growth is the same. Therefore, *S. proteamaculans* were used in experiments both after 24 h of growth and after 48 h of growth. The intensity of *S. grimesii* invasion decreases at 48 h of growth. Therefore, *S. grimesii* were used in experiments only after 24 h of growth. For the experiment, the bacteria were grown to the stationary growth phase, and the number of bacteria was determined using optical density at 600 nm. Aliquots containing an equal number of bacteria were taken from each bacterial suspension. 

### 4.2. Quantitative Invasion Assay

The efficiency of invasion was evaluated using the quantitative invasion assay [53,54]. Cells forming a 50–70% monolayer were pretreated with the 5 mM methyl-β-cyclodextrin (Sigma-Aldrich, Munich, Germany) (MβCD) for the indicated time or with 30 μM tyrphostin AG-1478 (Sigma-Aldrich, Munich, Germany) (stock solution 30 mM in ethanol) for 30 min followed by washing with DMEM. After MβCD and tyrphostin AG-1478 treatment, the proportions of viable cells were measured after staining with trypan blue through counting in a Goryaev chamber. Incubation with MβCD for 2 h and tyrphostin AG-1478 for 30 min did not affect the viability of eukaryotic cells. *S. grimesii* and *S. proteamaculans* were grown in LB medium for 24 h. Bacteria were pelleted at 13,000 rpm for 5 min; the pellets were resuspended in DMEM and added to host cells in a fresh portion of DMEM at a ratio of 100 bacteria per cell. Bacteria were deposited on the surface of the host cell using centrifugation for 5 min at 2000 rpm. After co-cultivating host cells and bacteria at 37 °C in 5% CO_2_ for 2 h, unattached bacteria were washed out twice with PBS, and the infected cells were suspended in 0.25% trypsin-versene solution. To quantify the effectiveness of invasion, the suspension of infected cells was incubated with an equal volume of DMEM medium containing 100 μg/mL gentamicin for 1 h at 37 °C to kill extracellular bacteria; then, cells were lysed with 1.5% sodium deoxycholate, quickly diluted with cold LB medium, and aliquots of the resulting suspension were plated on LB agar to determine the number of colony forming units (CFU) of the intracellular bacteria. The results for each experiment were the average of an assay performed in triplicate and independently repeated three times.

### 4.3. Fluorescence Microscopy

Cells forming a 50–70% monolayer on coverslips were pretreated with the 5 mM methyl-β-cyclodextrin (Sigma-Aldrich, Munich, Germany) for 30, 60 or 120 min. Cells were washed three times with PBS solution at each staining step. The preparations were fixed with 3.7% formaldehyde solution (Sigma-Aldrich, Munich, Germany) for 10 min. For labelling the lipid rafts, cells were stained with cholera toxin subunit B conjugated with FITC (Sigma-Aldrich, Munich, Germany) for 15 min at 4 °C. To visualize the actin cytoskeleton, the cells were incubated with 0.1% Triton X-100 for 5 min and stained with rhodamine-phalloidin for 15 min at 37 °C. To visualize the DNA of the epithelial cells, the cells were stained with DAPI for 5 min. The preparations were analyzed using an Olympus FV3000 microscope (Olympus, Tokyo, Japan) with a system of lasers with wavelengths of 405 (blue fluorescence), 488 (green fluorescence) and 561 nm (red fluorescence).

### 4.4. siRNA Transfection

The expression of host cell proteins was inhibited using siRNA targeting EGFR (sc-29301) (Santa Cruz, Heidelberg, Germany). Transfection of siRNAs was performed using a siRNA Transfection Reagent (sc-29528) as recommended by the manufacturer (Santa Cruz, Heidelberg, Germany). The RNA interference efficiency was controlled through real-time RT-PCR and Western blotting as shown before [29].

### 4.5. Western Blot Analysis

After transfection of siRNAs, cells were incubated with electrophoresis sample buffer (4% SDS, 24% glycerol, 200 mM DTT, 0.01% bromphenol blue, 125 mM Tris-HCl, pH 6.8) for 5 min at 56 °C. Cells were scraped off the plate, followed by a 5 min boiling. The samples were fractionated by SDS-PAGE and transferred to a Hybond ECL membrane according to the manufacturer’s instructions (GE Healthcare, Chicago, IL, USA). The membrane was incubated with 5% nonfat milk in PBS 40 min to prevent nonspecific binding of antibodies and then incubated with rabbit primary antibodies against EGFR [E235] at a dilution of 1:1000 (Abcam, Cambridge, UK) at room temperature for 1 h. The membrane was then washed three times with washing buffer (5% nonfat milk, 0.1% Tween 20, PBS) for 10 min, incubated for 2 h with the secondary antibodies (1:20,000) against rabbit IgG conjugated with horseradish peroxidase. The blots were washed with washing buffer three times and developed using SuperSignal West FEMTO Chemiluminescent Substrate (ThermoFisher Scientific, Waltham, MA, USA) according to the manufacturer’s recommendations.

### 4.6. Real-Time RT-PCR

Cells forming a 70–90% monolayer were pretreated with the 5 mM methyl-β-cyclodextrin (Sigma-Aldrich, Munich, Germany) for 30 or 120 min. Total RNA was extracted from cells using the Dia-M Extraction Kit according to the manufacturer’s instructions (Dia-M, Moscow, Russia). Reverse transcription and amplification were performed with the BioMaster HS-qPCR SYBR Blue (Biolabmix, Novosibirsk, Russia) using the CFX96 Touch Real-Time PCR machine (Bio-Rad, Irvine, CA, USA).

The steps included initial cDNA synthesis at 45 °C for 30 min, denaturation at 95 °C for 5 min, and 40 cycles of 95 °C for 30 s, 60 °C for 30 s and 72 °C for 30 s. Each sample was run in triplicate. Target gene expression was normalized to the expression of a cellular housekeeping gene, β-actin and GADPH, and calculated using the 2^−ΔΔCT^ method. The gene-specific primer pairs (Evrogen, Moscow, Russia) designed using BLAST-primer software and used for real-time PCR are listed in Table 1.

### 4.7. Statistical Analysis

Each quantitative experiment was repeated at least three times. Data were analyzed statistically using one-way analysis of variance (ANOVA) with an Excel Data Analysis Pack. A difference was considered significant at the *p* < 0.05 level.

## Figures and Tables

**Table 1:** Gene-specific primer pairs.

Target Gene	Primer Sequences
EGFR	Forward 5′-GTGCAGCTTCAGGACCACAA-3′
	Reverse 5′-AAATGCATGTGTCGAATATCTTGAG-3′
β-actin	Forward 5′-AATCTGGCACCACACCTTCTACA-3′
	Reverse 5′-GACGTAGCACAGCTTCTCGTTA-3′
GADPH	Forward 5′-GGCATGGACTGTGGTCATGAG-3′
	Reverse 5′-TGCACCACCAACTGCTTAGC-3′

## Supplementary Materials

The following supporting information can be downloaded at: https://www.mdpi.com/1422-0067/24/10/9029/s1.
