# Supplementary material for: Involvement of Lipid Rafts in the Invasion of Opportunistic Bacteria Serratia into Eukaryotic Cells
Source: Int J Mol Sci. 2023 May 20;24(10):9029. doi: 10.3390/ijms24109029 (PMC10361209; doi:10.3390/ijms24109029)
Supplement: Supplementary file 1 [file ijms-24-09029-s001.zip › ijms-2396250-supplementary.pdf]

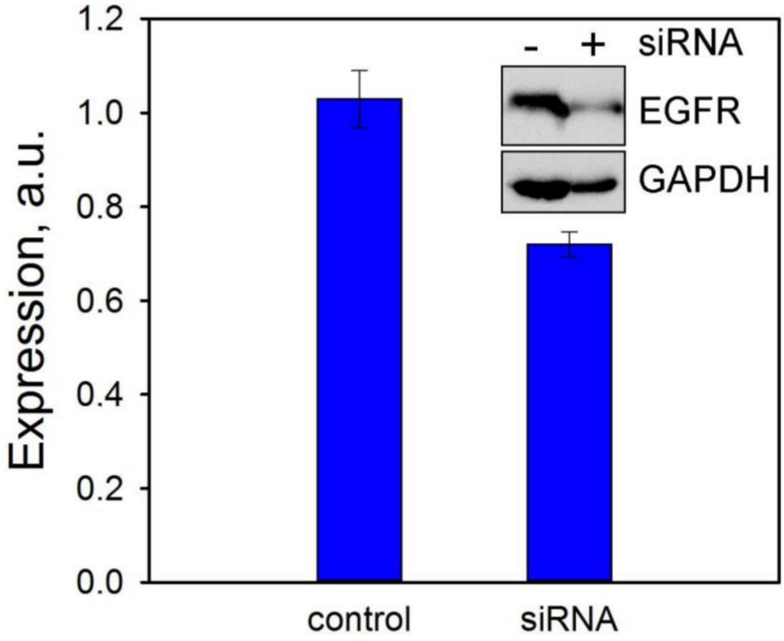

Figure S1. **Effect of treating M-HeLa cells with siRNA on EGFR expression in the host cell.** Expression levels of EGFR in treating M-HeLa cells with siRNA targeting EGFR were determined using real-time RT-PCR. Control - M-HeLa cells transfected with siRNA containing scrambled nucleotide sequence. The insert shows the total amount of EGFR and internal control GAPDH in untreated M-HeLa cells and pretreating cells with small interfering RNA at the according to the manufacturer’s protocol. Values are expressed as mean ± S.D. (error bars). A difference was considered significant at the $p<0,05$ level.
